# CRISPR/Cas9-targeting of CD40 in hematopoietic stem cells limits immune activation mediated by anti-CD40

**DOI:** 10.1371/journal.pone.0228221

**Published:** 2020-03-10

**Authors:** Rui Wang, Sean Graham, Ning Sun, Donna McCarthy, Ruoqi Peng, Jamie Erickson, Liz Oconnor, Xiaochun Zhu, Marc Wurbel, Robert Dunstan, Susan Westmoreland, Namjin Chung, Tariq Ghayur, Jijie Gu

**Affiliations:** 1 Abbvie, Cambridge Research Center, Cambridge, Massachusetts, United States of America; 2 AbbVie Bioresearch Center, Worcester, Massachusetts, United States of America; 3 AbbVie Inc., North Chicago, Illinois, United States of America; Harvard Medical School, UNITED STATES

## Abstract

Inflammatory bowel diseases (IBD) are complex, multifactorial disorders characterized by chronic relapsing intestinal inflammation. IBD is diagnosed around 1 in 1000 individuals in Western countries with globally increasing incident rates. Association studies have identified hundreds of genes that are linked to IBD and potentially regulate its pathology. The further dissection of the genetic network underlining IBD pathogenesis and pathophysiology is hindered by the limited capacity to functionally characterize each genetic association, including generating knockout animal models for every associated gene. Cutting-edge CRISPR/Cas9-based technology may transform the field of IBD research by efficiently and effectively introducing genetic alterations. In the present study, we used CRISPR/Cas9-based technologies to genetically modify hematopoietic stem cells. Through cell sorting and bone marrow transplantation, we established a system to knock out target gene expression by over 90% in the immune system of reconstituted animals. Using a CD40-mediated colitis model, we further validated our CRISPR/Cas9-based platform for investigating gene function in experimental IBD. In doing so, we developed a model system that delivers genetically modified mice in a manner much faster than conventional methodology, significantly reducing the time from target identification to *in vivo* target validation and expediting drug development.

## Introduction

Inflammatory bowel disease (IBD) refers to conditions in which patients’ immune system attacks their own digestive system, resulting in chronic inflammation in all or part of the gastrointestinal tract. In the US, approximately 1.5 million people currently suffer from IBD. It can be a devastating disease as it is progressive, persistent and relapsing without curative treatment, often resulting in repeated surgeries for patients throughout life. The lack of effective treatment for IBD represents a tremendous unmet medical need. The development of novel treatment and prevention strategies requires deciphering of the underlying mechanisms of the disease initiation and progression [1, 2].

Genome-wide association analyses (GWAS) have successfully identified over 230 different IBD loci [3] and shed light on the mechanisms and pathways important for IBD development. However, only a limited number of genes from those loci have been characterized. These include: the NOD2 microbe sensing pathway; autophagy pathway highlighted by ATG16L1, IRGM and CARD9; proinflammatory pathways such as IL-23-driven T cell responses [3]. Experimental animal models have been employed to functionally characterize GWAS-associated genes in disease development [4, 5]. Among the models for studying IBD pathogenesis [6, 7], injection of an anti-CD40 agonist antibody into T and B cell-deficient animals induces innate immune inflammation in the colon. This model is often used to investigate the roles of macrophages and dendritic cells in IBD development [8]. Gene-specific tools are also critical to characterize gene function in animal models, including chemical compounds, biological agents, and more importantly, genetically modified animals. Traditional strategies making genetic alterations in mice involve embryo injection and animal breeding, which often takes over a year to generate a strain with the desired genetic modification. This time-consuming process poses significant challenges to timely delineating the functions of all IBD GWAS-associated genes. A whole-body knockout is also not feasible for targets that are embryonic lethal.

To address those gaps, we employed the CRISPR/Cas9-based genome editing system, which allows rapid genetic manipulation [9–13], to accelerate animal model generation. CRISPR/Cas9-based edits have been introduced into adult animals using multiple approaches, including hydrodynamic injection, cationic lipid-mediated delivery, and adeno-associated virus (AAV)-mediated delivery [13–17]. These approaches, however, only deliver the CRISPR/Cas9 machinery to certain regions within the animals’ body and have limited applications. In the current study, we used CRISPR/Cas9 to edit hematopoietic stem cells (HSCs), also known as Lin-Sca1+Kit+ cells (LSKs) in mice, and tested our hypothesis that by combining the CRISPR technology and LSK transplantation, we could introduce genetic alterations in the entire immune system of the reconstituted animals. Although LSKs have been used to deliver CRISRP/Cas9 into recipient animals previously [18, 19], none of the existing literature enriched genetic modified LSKs, therefore limiting the application to phenotypic screens. Using CD40 as a model target, we knocked out this key regulator through CRISPR/Cas9-based editing and validated the loss of CD40 gene function in the CD40 agonist-induced colitis model. We demonstrated for the first time that protein expression can be reduced by 90% or higher in the immune system within four months, which is significantly shorter than the time traditional method needs to introduce systemic genetic alterations *in vivo*, and meets the need for a fast and efficient system to functionally characterize IBD-associated genes in preclinical models. As such, the method showcased in this study provides a step forward regarding the use of CRISPR/Cas9 technology in biomedical research and may benefit drug discovery for not only IBD but also other autoimmune diseases.

## Materials and methods

### Mice

The Cas9 Knockin mice (stock number 026179), C57BL/6-Ly5.1 (stock number 002014) and C57BL/6-Ly5.2 (stock number 000664) mice were ordered from Jackson Labs (Bar Harbor, ME). Mice were used at 6–8 weeks of age. All mice were maintained in a specific pathogen-free facility and routinely checked for health conditions. Mice received bone marrow transplantation were housed in sterile cages with autoclaved bedding and food. Antibiotic were given to the drinking water for up to 6 weeks post-transplantation. Hydration supplement, such as H-gel, were also provided to alleviate discomfort. Animals were euthanized using CO_2_ prior to tissue collections. All studies involving animals were performed according to protocols reviewed and approved by the AbbVie Institutional Animal Care and Use Committee (IACUC).

### Plasmids construction

The puromycin resistant gene in the lentiGuide-Puro vector (Addgene, Watertown, MA; Plasmid number 52963) was changed to vexGFP or mCherry encoding gene. The scramble control guide RNA (SgNone), guide RNAs targeting CD44 [20] or CD40 were subsequently cloned into the vexGFP vector. The guide RNA (gRNA) sequences used in the present study are as follows: SgNone, CTATGATTGCAACTGTGCAG; CD44, TATGGTAACCGGTCCATCGA; SgCD40.1, AGCGAATCTCCCTGTTCCAC; SgCD40.2, GACAAACAGTACCTCCACGA; SgCD40.3, ACGTAACACACTGCCCTAGA

### Lentivirus production

Lentiviral particles were produced as described previously [21]. Briefly, gRNA-encoding plasmid was co-transfected into 293T cells together with VSV-G and pLEX packaging plasmids (Addgene). Culture medium was changed 18 hrs post-transfection, and the virus-containing supernatants were collected 24–48 hrs post-medium change. To concentrate the virus, the harvested supernatants were centrifuged at 34,000g for 90 min, and the pelleted virus was resuspended in 1–2% of the original supernatant volume.

### Knockout mice generation using LSK cells

LSK cells were isolated and infected as previously described [22]. Briefly, bone marrow cells were collected from femurs and tibias of adult animals, and red blood cells were lysed with lysis buffer (eBioscience, San Diego, CA). CD117 (c-kit)+ cells were isolated using CD117 magnetic beads (Miltenyi, Somerville, MA) and then sorted for Lin- and Sca1+ population using a BD Aria sorter (San Jose, CA). In some experiments (S1 Fig), 5-fluorouracil was injected at 150 mg/kg a week before LSK cell isolation. The pluripotency of the isolated LSK cells were confirmed using the Colony-Forming Unit (CFU) Assay according to the manufacturer’s instructions (STEMCELL Technologies, Vancouver, Canada). The LSK cells were rested overnight in SFEM medium (STEMCELL Technologies) with a mixture of cytokines (100 ng/ml of Fit3L, IL-7, SCF, TPO each; all from PeproTech, Rocky Hill, NJ) before transferred to Retronectin coated plates (50ug/ml; Clontech, Mountain View, CA). Lentivirus expressing gRNA or scramble control were added to the cells at a multiplicity of infection (MOI) of 50–100, and spun down at 700g for 20min. The infected cells were further cultured for 2 days before injected into the animals. Recipient animals were irradiated with gamma irradiation at 600 rads twice 3 hrs apart. Up to 50,000 LSK cells were intravenously injected into the recipient animals 3 hrs after the 2^nd^ dose of irradiation. After bone marrow transplant, mice were placed into autoclaved cages and treated as immune deficient animals for 12 weeks. To study the localization of donor-derived cells in the reconstituted animals, mCherry+ cells were sorted from the infected LSK cells and then injected to the recipients. The reconstituted animals were sacrificed at 12 weeks post-injection for tissue collection and analysis.

### *In vitro* efficiency evaluation of gRNAs

To evaluate CD44 gRNA efficiency *in vitro*, splenocytes from Cas9 Knockin mice were isolated and stimulated with 1μg/ml anti-CD3e (eBioscience) in the presence of viral particles expressing SgNone or SgCD44. The cells were expanded in the presence of 100U/ml IL-2 (PeproTech) for two weeks before stained for surface CD44 expression.

To evaluate CD40 gRNA efficiency, we generated a CD40 stable cell line by transfecting a pcDNA3.1 plasmid encoding murine CD40 into 293T cells. The cells were then infected by lentivirus expressing SgNone or gRNAs targeting CD40 (SgCD40.1, SgCD40.2 and SgCD40.3). Expression of CD40 was evaluated by FACS two weeks post-infection. One-way ANOVA with Dunnett’s post hoc test (vs SgNone control group) was performed to calculate statistical significance among animal groups using GraphPad Prism 5.0 (GraphPad Software, San Diego, CA). Each group contained 4–10 mice. P<0.05 was considered statistically significant.

### CD40-agonist induced colitis model

The CD40 agonist antibody FGK4.5 (BioXcell, West Lebanon, NH) was intraperitoneally injected at 10 mg/kg per animal on day 0. In some experiments (S3 Fig), anti-p40 antibody (clone C17.8, BioXcell) was injected at 10 mg/kg every other day starting from day -1. Body weight was monitored every day beginning day -1. Video colonoscopy on Day 3 and 6 following CD40 antibody injection was performed to evaluate disease progression as previously described [23]. Briefly, mice were first anesthetized with 2% isofluorane, and then given an enema using DPBS to prepare the colon for colonoscopy. The endoscope (Karl-Storz, Southbridge, MA) was inserted into the rectum, advanced to the proximal colon, and then slowly withdrawn while taking a video recording. Images were taken at 3, 2, and 1cm distal of the anus. A scoring system was developed to determine disease severity in each image. Scores from individual images were then combined to create a sum colonoscopy score for each animal. Vascular pattern was scored on a scale from 0 to 3; 0 = large/small vessels are bright, sharp, and have a continuous pattern, 1 = large/small vessels are visible but not connecting and out of focus, 2 = large vessels are still visible but discontinuous and a number of small vessels appear to have burst, 3 = no vessels are visible and the surface of the colon is very bumpy. Thickening of the mucosal surface was scored on a scale from 0 to 3: 0 = smooth and shiny surface; 1 = the mucosal wall is less transparent and slightly bumpy with a shiny mucous layer; 2 = clear white, shiny, bumpy layer covering most of the circumference; 3 = opaque white bumpy surface covering the circumference. On Day 7, all animals were euthanized, the colon was harvested for histopathological assessment, and the spleen was weighed then prepared for analysis by flow cytometry. To analyze statistical differences between control and CD40 knockout groups, One-way ANOVA’s with Dunnett’s post hoc test (vs SgNone control group) was performed using GraphPad Prism 5.0.

### Immunohistochemistry (IHC)

Formalin-fixed and paraffin-embedded (FFPE) tissue sections of mouse colon were used for IHC staining of mCherry, ionized calcium binding adaptor molecule 1 (IBA1) and CD3. Immunohistochemical staining was performed on a Leica Bond RX^®^ automated stainer (Leica Biosystems, Wetzlar, Germany) using the Bond Polymer Refine DAB Detection kit (Leica Biosystems) following the manufacturer’s instructions. The sections were subjected to ethylenediaminetetraacetic acid (EDTA) based antigen retrieval for 20 min followed by application of the primary antibody. To identify donor cells, anti-mCherry antibody (ThermoFisher Scientific, Waltham, MA) was used at 5μg/ml for 30min at room temperature, hematoxylin was used as a counterstain. To identify macrophages, antibody to IBA1 (Wako Chemicals USA, Richmond, VA) was used at 0.15μg/ml for 30 min at room temperature, and alcian blue was used as a counter stain to identify goblet cells. To identify T-lymphocytes, anti-CD3 antibody (Lab Vision, Fremont, CA) was used at 0.08μg/ml for 15 min at room temperature, and hematoxylin was used as a counterstain. Following staining, samples were digitized using a Pannoramic scanner (3DHistech, Hungary). Visiopharm software (Denmark, Germany) was used for image analysis. This was performed by hand annotating a 150 mm bounding box in the mid colon in each sample. For the IBA1/alcian blue stained sections, an algorithm was written to define the mucosal area, the area of IBA1+ staining and the area of goblet cell+ staining. Mucosal thickness in the mid colon region in the bounding box was defined by dividing the mucosal area by 150 mm. The IBA1+ and goblet cell+ staining was expressed as % of total mucosal area. A third algorithm using a similar method was used to define the CD3+ stain as % of total mucosal area.

### FACS

Tissues including spleen, bone marrow and blood were isolated from animals, and cells were extracted from the tissues. Red blood cells were lysed by lysis buffer (eBioscience) and the cells were stained using fixable live/dead dye (ThermoFisher Scientific) following the manufacturer’s instructions. The cells were then stained with antibodies of interest at 4°C for 20 min. Cells were washed with FACS buffer and analyzed with a BD LSRFortessa^™^ cell analyzer. Antibodies used include B220, GR-1, Ter119, CD11b, CD3, CD40, CD44, CD45.1, CD45.2, Thy1.1, and Sca-1 (all from Biologend, San Diego, CA).

## Results

### Establishment of a transplantation protocol using LSK cells

**Fig 1:**
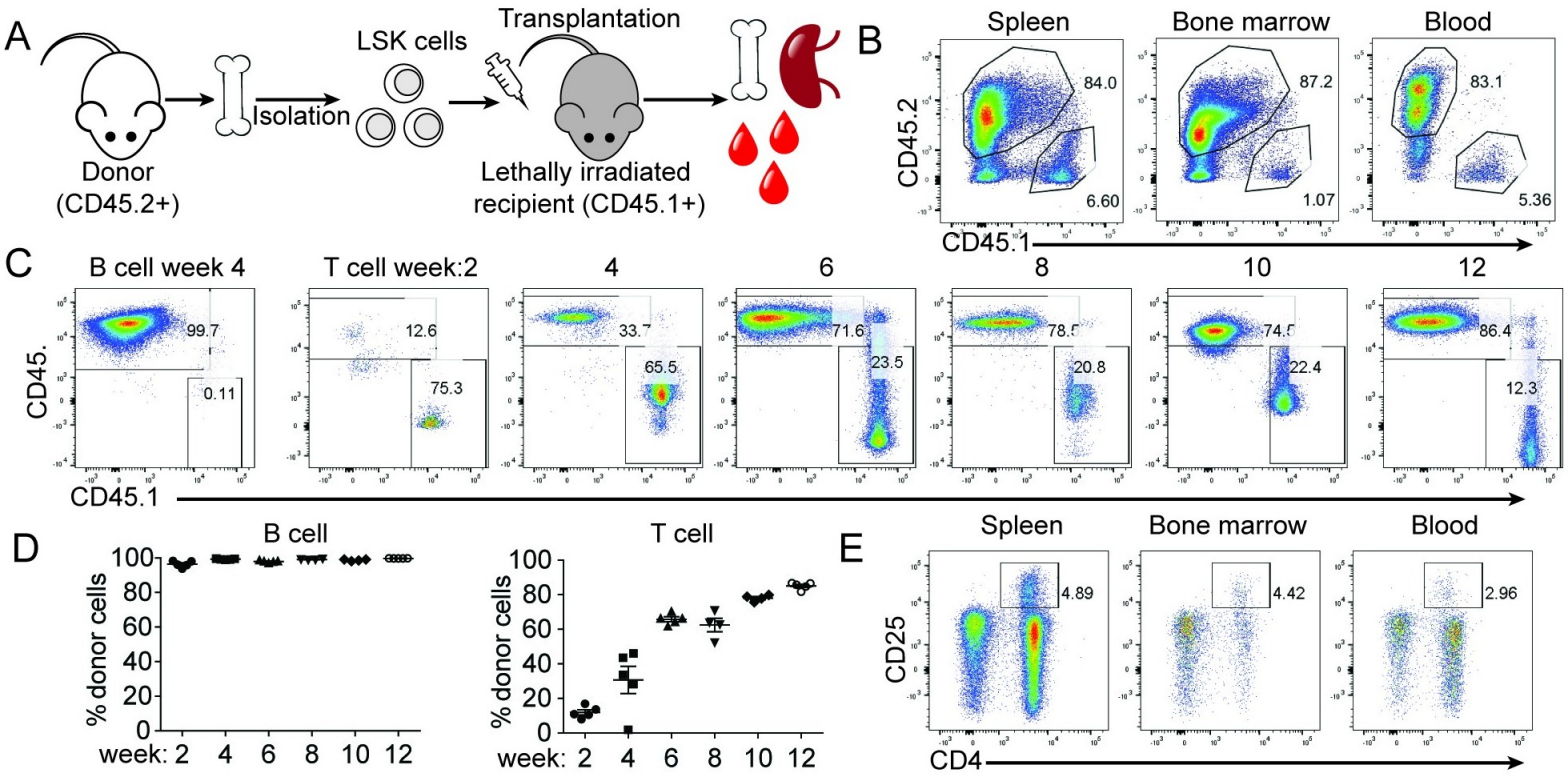
Establishment of a transplantation protocol using LSK cells. (A) Experimental setup. LSK cells were isolated from CD45.2+ donor mice and transplanted to lethally irradiated CD45.1 congenic C57BL/6 animals. Five mice were taken down every other week post-transplantation, and the engraftment rates in the spleen, bone marrow and blood were evaluated by FACS. (B) Representative FACS plots in the spleen, bone marrow and blood at Week 2 post-transplantation. (C) Representative FACS plots of B cells at Week 4 and T cells at different timepoints post-transplantation. (D) Percent of donor B and T cells at different timepoints post-transplantation. Each dot represents a data point from a single animal. (E) Representative Treg population in spleen, bone marrow and blood at Week 12 post-transplantation. Data shown are representative of two independent experiment.

We first established a protocol to reconstitute the immune system of irradiated animals using LSK cells (Fig 1A). We initially attempted to enrich LSK cells by pre-treating the donor animals with 5-fluorouracil, and found that c-kit expression on the enriched Lin-Sca1+ cells markedly decreased (S1A Fig), which was consistent with prior findings showing that 5-fluorouracil treatment led to reduced c-kit expression [24, 25]. Therefore, we isolated LSK cells following a two-step sorting as previously described [22]. We first isolated c-kit+ cells from bone marrow with magnetic cell sorting, and then further purified Lin-Sca1+ cells with FACS sorting. Sorted CD45.2+ cells were injected into lethally irradiated CD45.1+ congenic recipients. CD45.2 and CD45.1 were used as markers to identify the donor and recipient cells, respectively. Prior to transplantation, the quality of sorted LSK cells was confirmed *in vitro* using a commercial mouse CFU Assay, which measures the proliferation and differentiation ability of individual HSCs by assessing the colonies produced by each progenitor cell. To monitor engraftment, five mice were taken down every other week post-transplantation. As shown in Fig 1B, we achieved over 80% engraftment in the spleen, bone marrow, and blood at Week 2 post-transplantation. To examine lineage maturation, we performed a detailed characterization of different immune cell types. B cells, CD11b+ macrophages, and CD11c+ dendritic cells (DCs) were fully engrafted at Week 2 (Fig 1C and 1D, S1B Fig). In contrast, the T cell compartment required a minimum of 12 weeks to reach approximate 90% engraftment (Fig 1C and 1D). This observation agreed with the previous finding that T cells need to undergo thymic selection to fully mature [26]. To ensure proper hematopoiesis in the reconstituted animals, we validated the generation of regulatory T cells (Tregs) and neutrophils within the T and myeloid populations by CD25 and Ly6G staining, respectively (Fig 1E and S1C Fig). We further validated that the proportion of Tregs generated in this system resembled those from WT mice (S1D Fig). Taken together, we have established a protocol to reconstitute the murine immune system *in vivo* by transplanting LSK cells into lethally irradiated recipient animals.

### Lentiviral infection of LSK cells and analysis of donor-derived cells in reconstituted mice

**Fig 2:**
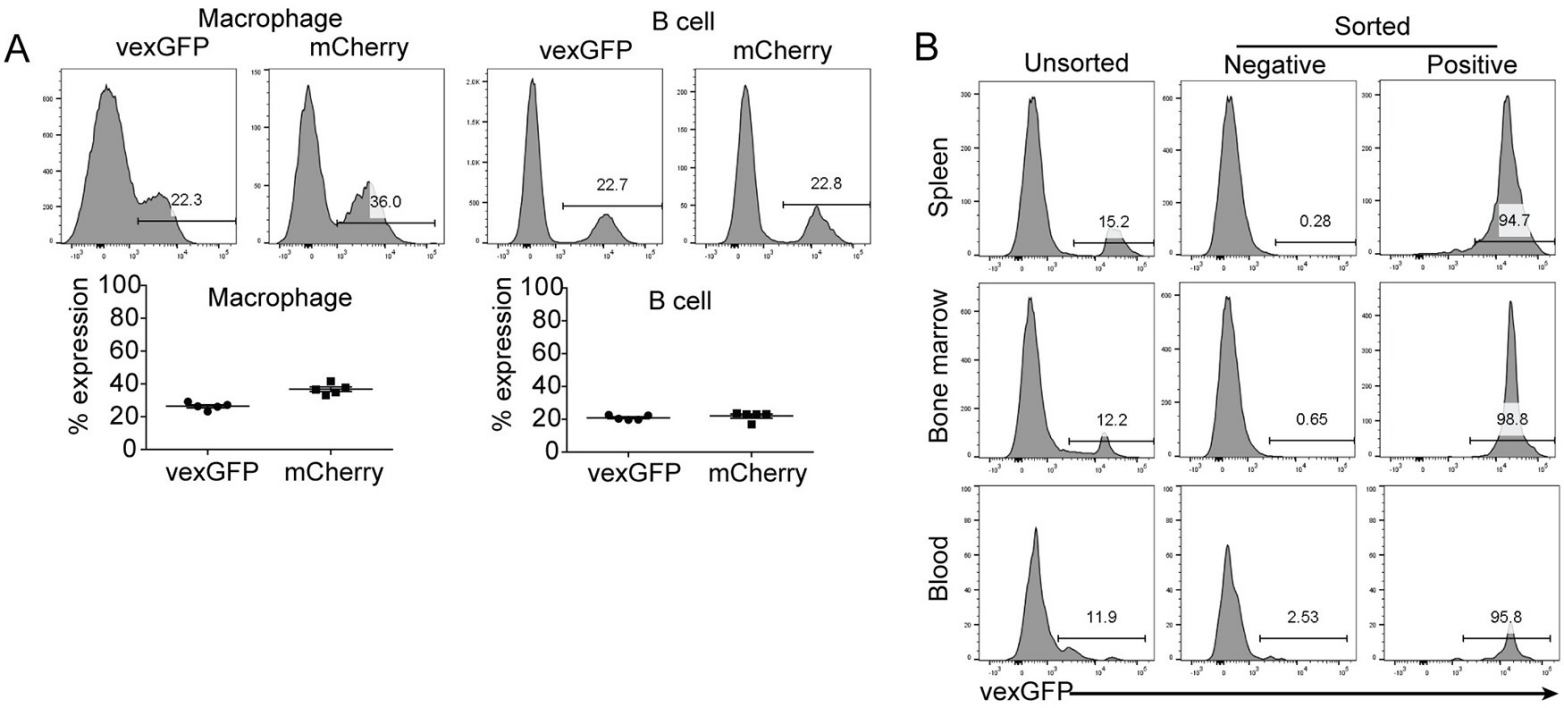
Lentiviral infection of LSK cells. (A) Reporter gene expression in reconstituted mice. Mice were transplanted with LSK cells infected with vexGFP- or mCherry-expressing lentivirus. Upper panel, representative FACS plots showing vexGFP and mCherry expression in macrophages and B cells. Lower panel, percent of vexGFP+ and mCherry+ cells from reconstituted mice. Each dot represents a single animal. (B) Isolation of infected LSK cells. LSK cells were infected by virus expressing vexGFP at a MOI of 1 and sorted for vexGFP expression. Unsorted cells, vexGFP+ and vexGFP- cells were injected into recipients (five animals per group). Mice were harvested 3 weeks post-reconstitution. Depicted are representative FACS plots from each group. Data shown are representative from two independent experiments.

Next, we infected LSK cells with lentivirus expressing vexGFP or mCherry and engrafted the infected cells to lethally irradiated recipient mice. The expression of both vexGFP and mCherry was detected three weeks post-transplantation from all tissues examined including the spleen, blood and bone marrow (Fig 2A), suggesting that both vexGFP and mCherry can be used to track infected cells *in vivo*. Both vexGFP+ and vexGFP- donor cells differentiated into similar proportions of macrophage and B cells (S1E Fig), suggesting that lentiviral infection and vexGFP expression did not interfere with hematopoiesis of donor LSK cells.

To further optimize the transplantation platform and generate animals with more homogenous knockout of the immune system, we further purified infected cells based on reporter gene expression. LSK cells were infected with vexGFP-expressing virus at a MOI of 1, and then sorted for vexGFP expression. Over 90% of immune cells expressed vexGFP in mice reconstituted with vexGFP+ LSK donor cells (Fig 2B).

To further characterize donor cell differentiation in the recipient animals, we used mCherry to track donor-derived cells in the reconstituted mice. As expected, donor-derived cells were most abundant in the immune organs including spleen and lymph nodes, but were also present in other organs including the lung, liver and intestine (S2 Fig). Taken together, we successfully established a platform to lentivirally infect LSK cells and enrich the infected LSK cells for immune system reconstitution *in vivo*.

### Reduction of CD44 expression *in vivo* using CRISPR/Cas9-based technology and LSK transplantation

**Fig 3:**
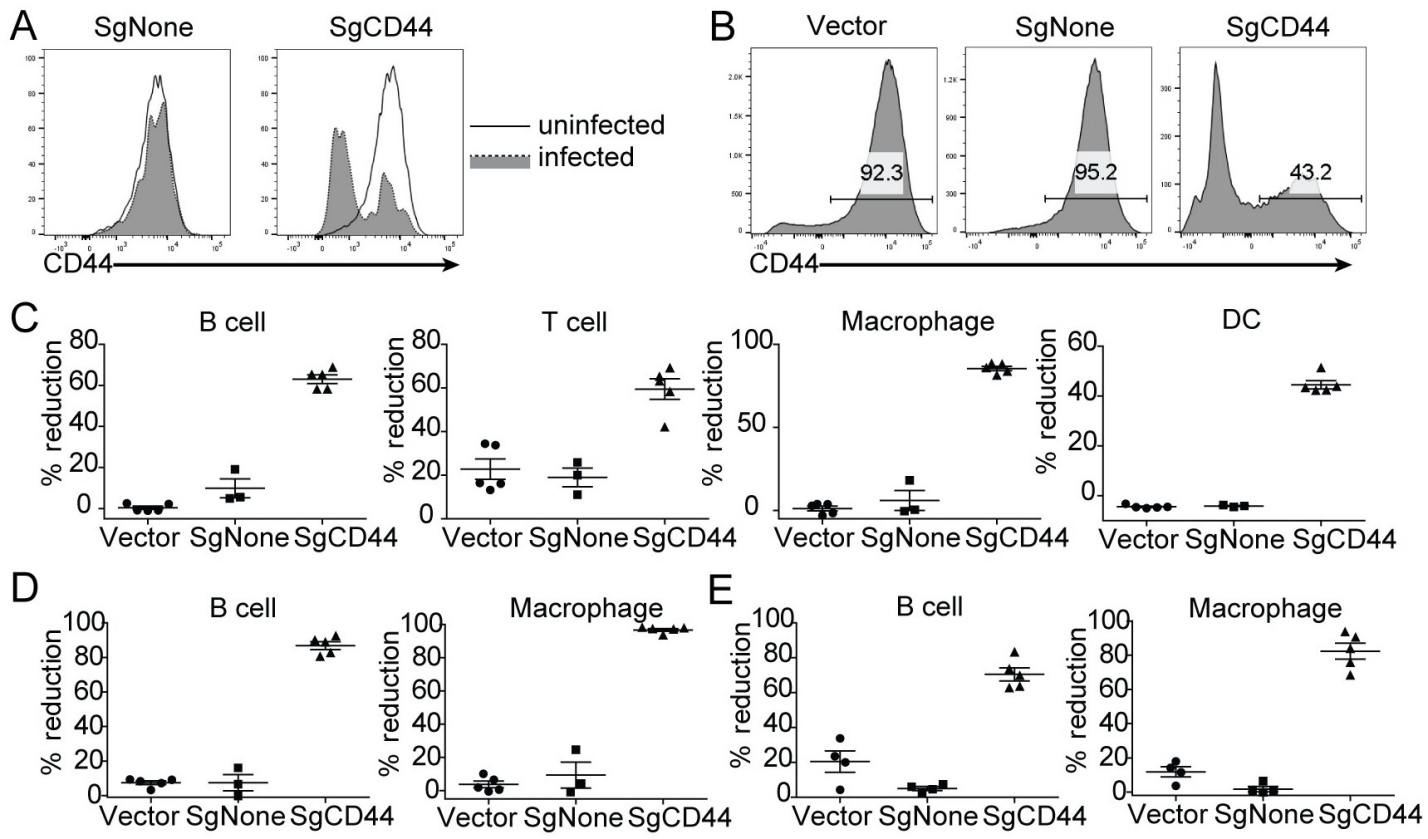
Modulation of CD44 expression using CRISPR/Cas9-based system. (A) Downregulation of CD44 expression *in vitro*. Splenocytes were isolated from Cas9 Knockin mice, and infected with viruses expressing SgNone or SgCD44. (B, C, D) Downregulation of CD44 expression *in vivo*. Mice were generated to express the vector control, SgNone or SgCD44. (B) Representative FACS plots showing CD44 expression from splenic B cells. (C) Reduction of CD44 expression in B cell, T cell, macrophages and DCs in the spleen. (D and E) Reduction of CD44 expression in B cells and macrophages from bone marrow (D) and blood (E). Each dot represents an individual animal.

Next, we chose to use CD44, a marker gene expressed on various subsets of immune cells, to evaluate the editing efficiency of our platform. After *in vitro* validating the efficacy of a previously published [20] gRNA targeting CD44 (SgCD44) (Fig 3A), we infected LSK cells from Cas9 Knockin mice [27] with lentivirus expressing empty vector, scramble control (SgNone) or SgCD44, and engrafted the infected LSKs into CD45.1+ recipient mice. At eight weeks post-transplantation, cells expressing SgCD44, but not empty vector or SgNone, showed a strong reduction of CD44 expression in the spleen, bone marrow and blood (Fig 3B–3D). Thus, we have established an *in vivo* CRISPR/Cas9-based system for editing genes of interest in immune cells.

### CRISPR/Cas9-based knockout of CD40 ameliorates disease pathogenesis in a CD40 agonist-induced colitis model

**Fig 4:**
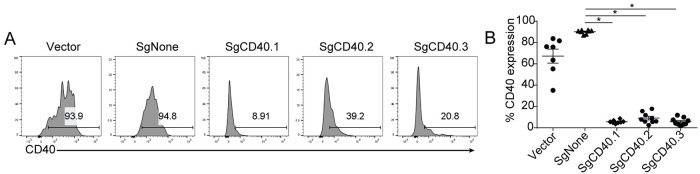
Modulation of CD40 expression using CRISPR/Cas9-based system. (A) CRISPR/Cas9-mediated reduction of CD40 expression in 293T cells stably expressing CD40. (B) CRISPR/Cas9-mediated reduction of CD40 expression in the reconstituted mice. Splenic B cell CD40 expression was evaluated 8 weeks post-transplantation. Each dot represents an individual animal. * = P<0.005 Data shown are representative of two independent experiments.

Next, we tested our CRISPR/Cas9-based knockout strategy in an experimental model of IBD. The CD40 pathway has been associated with several types of autoimmune diseases including colitis, arthritis and lupus [28]. CD40 agonist antibodies are known to induce systemic inflammation and colitis in immune deficient mice [8]. We hypothesized that knocking out CD40 using our CRISPR/Cas9-based platform would prevent disease development in the CD40 agonist-induced colitis model. To test this hypothesis, we designed three different gRNAs that target mouse CD40 (SgCD40.1, SgCD40.2, and SgCD40.3) and validated their editing efficiency in CD40 expressing 293T cells (Fig 4A). We then introduced these gRNA *in vivo* using our LSK transplantation platform and showed that all three gRNAs achieved approximately 90% efficiency in reducing CD40 expression in splenic B cells (Fig 4B).

Since the anti-CD40 agonist antibody had only been shown to induce colitis in T and B deficient strains [8], we next confirmed that it also induced intestinal inflammation in WT C57BL/6 mice. The administration of anti-CD40 agonist antibody in WT C57BL/6J mice led to body weight loss, vasculature reduction shown by colonoscopy, and induction of myeloid cells infiltration shown by IBA1 IHC (S3 Fig). We also detected adaptive immune responses: T cell infiltration in the colon shown by CD3 IHC; T and B cell activation in the spleen shown by surface CD86 upregulation. In addition, anti-p40 blocking antibody effectively inhibited disease induction (S3 Fig), similar to what’s observed in T and B cell deficient strains [8].

**Fig 5:**
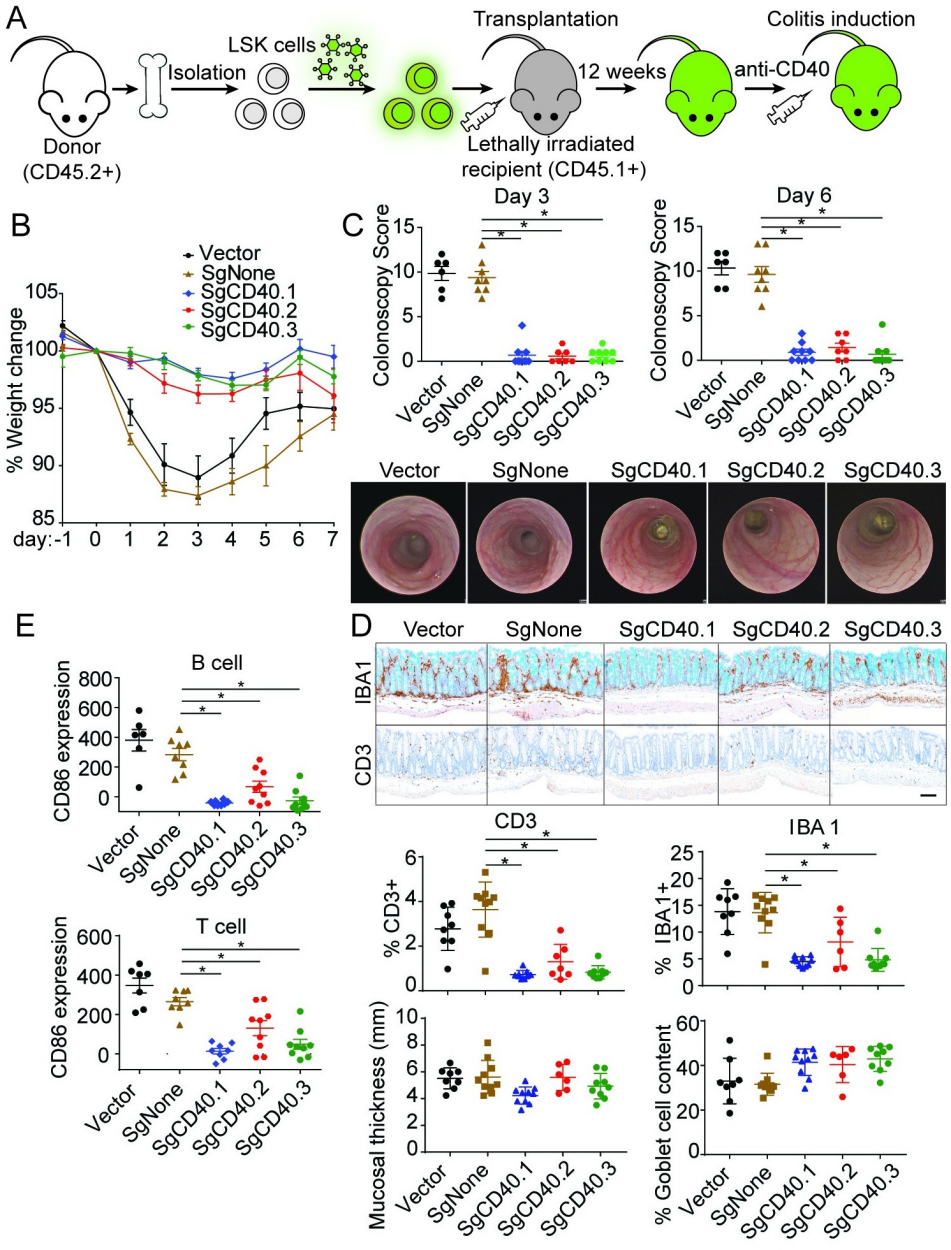
CRISPR/Cas9-based knockout of CD40 ameliorates disease pathogenesis in a CD40 agonist-induced colitis. (A) Experimental setup. LSK cells were infected with lentivirus expressing control or SgCD40 and sorted for vexGFP. VexGFP+ cells were used to transplant lethally irradiated CD45.1+ recipient mice (n = 10). 12 weeks post-transplantation, colitis was induced by injecting CD40 agonist antibody. (B–E) Disease induction was assessed by body weight change (B), colonoscopy at Day 3 and Day 6 post-anti-CD40 injection (C), percent of IBA1+ and CD3+ cell area of total mucosal area, mucosal thickness as well as percent of goblet cell area of total mucosal area (D), and upregulation of CD86 expression in splenic B and T cells (E). Representative Day 6 colonoscopy images (C) and Day 7 histology images (D) are included. Each dot represents an individual animal. Scale bar, 100μM. * = P<0.001. Data shown are representative of two experiments.

We next injected the anti-CD40 agonist antibody into the mice reconstituted with LSK cells expressing control or CD40 gRNAs. Disease severity was evaluated using multiple parameters, including weight loss, colonoscopy, upregulation of CD86 as a marker for T and B cell activation, and CD3 and IBA1 staining marking T and myeloid cell infiltration in the colon. Mice reconstituted with SgCD40-expressing LSK cells exhibited much milder colitis symptoms. All three gRNAs protected the animals from weight loss and clinical manifestations as measured by colonoscopy (Fig 5B and 5C). Furthermore, there was a clear correlation between the gRNA’s *in vitro* editing efficiency and its ability to inhibit disease induction (Fig 4A): SgCD40.1 showed the highest efficiency in reducing CD40 expression (Fig 4A), and led to the most robust inhibition of disease initiation shown by CD86 upregulation and immune cell infiltration (Fig 5D and 5E); SgCD40.2 was the least efficient gRNA among the 3 in reducing CD40 expression *in vitro* and also showed least protection *in vivo*; SgCD40.3 demonstrated intermediate efficiency both in reducing CD40 expression and in protecting mice from developing disease (Fig 5D and 5E). Taken together, these data demonstrated that CRISPR/Cas9-induced ablation of CD40 expression could effectively protect animals from colitis induction, and more importantly, our *in vivo* CRISPR/Cas9-based platform could be used to characterize gene function in colitis pathogenesis.

## Discussion

The work presented here introduces a novel system to study gene function *in vivo* using CRISPR/Cas9-based genome editing technology. By enriching genetically modified LSK cells, we achieved over 90% protein expression reduction in the immune cells of transplanted animals within four months. We further demonstrated that the reduction of CD40 expression in the immune system effectively prevented disease induction in the anti-CD40 agonist antibody-induced colitis model. Therefore, we have established a platform that enables efficient and effective characterization of gene functions in the immune system *in vivo*, which will significantly accelerate the validation of new target biology, and ultimately benefit patients with IBD and other autoimmune diseases.

We first established an LSK infection and transplantation protocol. Using our protocol, we observed proper differentiation of macrophage, DC, B and T cells, as well as cell subsets including Treg and neutrophils, in the reconstituted mice. We also validated that lentiviral infection and fluorescent marker gene expression exerted no significant impact on immune cell differentiation in those mice. We further investigated the localization of donor-derived cells in the reconstituted mice. Using mCherry as a reporter gene, we found a substantial number of donor-differentiated cells in the immune organs including spleen and lymph nodes. Although to a much lesser extent, we also found mCherry+ cells in tissues including lung, liver, intestine, kidney and skin. These findings improve our understanding of the LSK transplantation model and pave the way for more application of this model in biomedical research.

Our platform enables modulation of gene expression in adult mice in an efficient and effective manner. Several different approaches have been previously used to induce genetic alterations in LSK cells, including RNP-based and AAV-based approaches [29–31]. Gundry, et al. successfully edited genes in both human and murine HSCs with RNP-based approach [30], however, that study were limited to ex vivo analysis with a relatively low efficiency of 60%. *In vivo* engraftment studies have also been conducted in mice using AAV-transduced LSK cells [32], yet the efficiency was low and CRISPR/Cas9-based editing has not been evaluated using this system in LSK cells. We used a lentiviral system with CRISPR/Cas9-based technology to ablate gene expression, and further enriched infected LSK cells based on reporter gene expression. Leveraging these methods, we achieved over 90% gene knockout efficiency in the immune system of the reconstituted animals. Since it takes approximately twelve weeks for LSKs to repopulate the immune system in the reconstituted mice, we were able to generate mice with edited immune system within four months. Additionally, by targeting only the immune system, our platform may allow the *in vivo* analyses of target genes that are critical for embryonic development, if knockouts of those genes are lethal embryonically but viable in LSK reconstituted animals. Therefore, our approach is much faster than traditional methods to validate target biology in the immune system *in vivo*.

We further validated our platform for assessing gene function *in vivo*. Agonizing CD40 by antibodies induced intestinal inflammation in T and B cell deficient animals [8], but it was unclear whether it induced similar phenotype in WT mice. We first addressed this question by injecting anti-CD40 agonist antibody into WT C57BL/6 mice. Like Rag and Scid mice, agonizing CD40 induced inflammation in WT mice shown by body weight loss and myeloid cell infiltration in the colon. We also observed activation of adaptive immune response shown by T and B cell activation in the spleen, and by T cell infiltration in the colon. Anti-p40 prophylactic treatment strongly inhibited disease induction suggesting that the myeloid population played an important role driving the inflammation. We next evaluated whether ablating CD40 expression using our CRISPR/Cas9 platform could prevent disease induction in the reconstituted mice. our results showed that CD40 gRNAs efficiently reduced CD40 expression *in vitro*, and strongly inhibited inflammation induction *in vivo*. All the three gRNAs targeting CD40 markedly reduced CD40 expression in B cells, and significantly ameliorated disease development measured by body weight loss and colonoscopy. *In vitro* editing efficiency of the three gRNAs also correlated nicely with the extent of disease reduction shown by immune cell activation and infiltration. Although in the current study we only examined the impact of gene editing in a colitis model, it is fair to anticipate that this platform can be equally applied to investigate gene function in other preclinical models of autoimmune diseases.

In summary, we have combined LSK transplantation and CRISPR/Cas9 based technology, and established an *in vivo* platform to efficiently ablate gene expression in the immune system. Since one may edit two or more genes in the LSK cells using CRISPR/Cas9 simultaneously, our platform potentially allows researchers to simultaneously target multiple genes in the reconstituted animals, providing the opportunity to assess digenic or polygenic effects observed in IBD patients. Most importantly, our platform can be used to rapidly characterize genes with genetic association and significantly accelerate the time span between basic research discoveries and therapeutic development.

## Supporting information

S1 Fig Optimization of LSK transplantation protocol. (A) Impact of 5-fluorouracil in LSK enrichment. Mice were treated with 5-fluorouracil a week before LSK cell isolation. (B) Percent of donor macrophage and DCs in reconstituted mice at different timepoints post-transplantation. Each dot represents an animal. (C) Neutrophil development in the spleen, bone marrow and blood from reconstituted mice at Week 12 post-transplantation. Shown are representative FACS plots. (D) Treg development in spleen, bone marrow and blood from adult WT mice. (E) B cell and macrophages development within vexGFP+ and vexGFP- population at Week 8 post-transplantation. Shown are representative results from two independent experiments.
(TIF)

S2 Fig Distribution of donor LSK-differentiated cells in reconstituted animals. Recipient animals were reconstituted using LSK cells infected with mCherry expressing virus. Tissues were harvested at Week 12 post-transplantation and mCherry expression (brown) was evaluated by IHC: (A) spleen (B) mesenteric lymph nodes (C) lung (D) liver (E) small intestine (F) large intestine (G) kidney (H) skin.
(TIF)

S3 Fig Anti-CD40 agonist-induced intestinal inflammation in C57BL/6 mice. Anti-CD40 agonist antibody was injected to C57BL/6 mice to induce inflammation, and disease induction was evaluated based on body weight change (A), colonoscopy at Day 3 and Day 6 post-anti-CD40 injection (B), percent of IBA1+ and CD3+ area of total mucosal area, mucosal thickness as well as percent of goblet cell area of total mucosal area (C), and upregulation of CD86 expression in splenic B and T cells (D). In (B), representative images for Day 6 colonoscopy are shown. In (C), representative images for Day 7 histology are shown. Scale bar, 100μM. * = P<0.001 Data are representative results from two independent experiments.
(TIF)
