# Supplementary figures and images for: CRISPR/Cas9-targeting of CD40 in hematopoietic stem cells limits immune activation mediated by anti-CD40
Source: PLoS One. 2020 Mar 10;15(3):e0228221. doi: 10.1371/journal.pone.0228221 (PMC7064223; doi:10.1371/journal.pone.0228221)

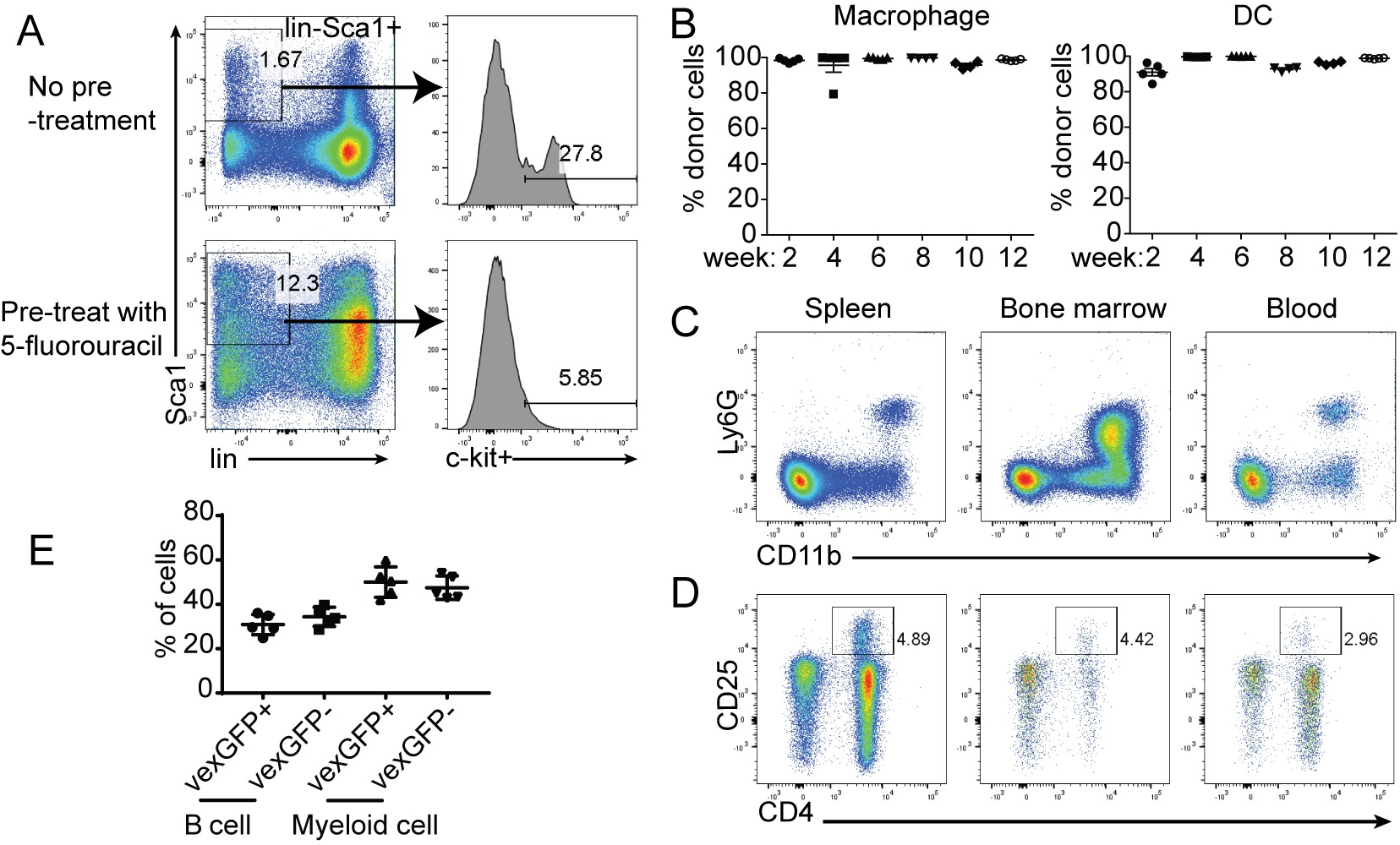

Supplement: S1 Fig — (A) Impact of 5-fluorouracil in LSK enrichment. Mice were treated with 5-fluorouracil a week before LSK cell isolation. (B) Percent of donor macrophage and DCs in reconstituted mice at different timepoints post-transplantation. Each dot represents an animal. (C) Neutrophil development in the spleen, bone marrow and blood from reconstituted mice at Week 12 post-transplantation. Shown are representative FACS plots. (D) Treg development in spleen, bone marrow and blood from adult WT mice. (E) B cell and macrophages development within vexGFP+ and vexGFP- population at Week 8 post-transplantation. Shown are representative results from two independent experiments. (TIF) [file pone.0228221.s001.tif]

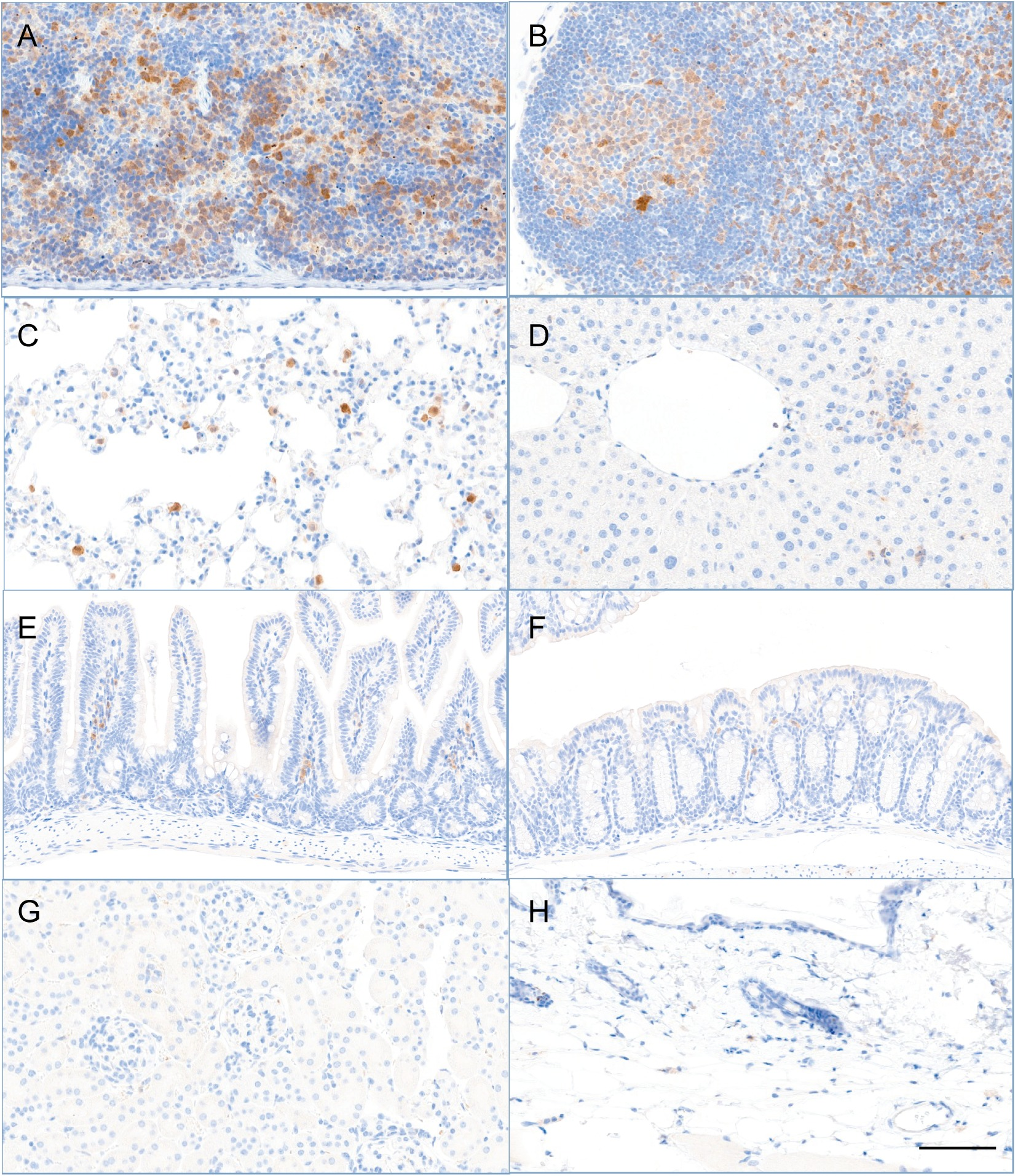

Supplement: S2 Fig — Recipient animals were reconstituted using LSK cells infected with mCherry expressing virus. Tissues were harvested at Week 12 post-transplantation and mCherry expression (brown) was evaluated by IHC: (A) spleen (B) mesenteric lymph nodes (C) lung (D) liver (E) small intestine (F) large intestine (G) kidney (H) skin. (TIF) [file pone.0228221.s002.tif]

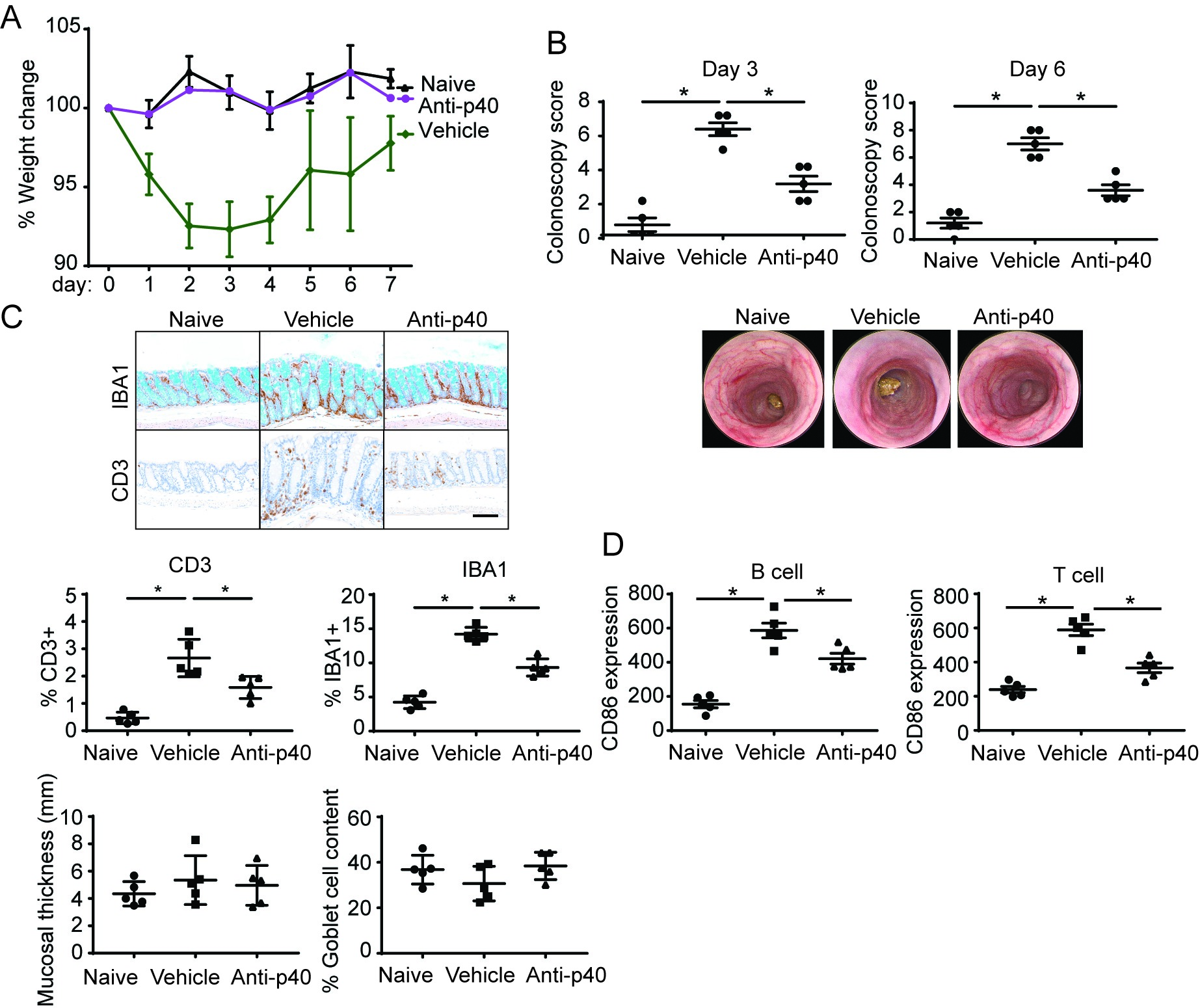

Supplement: S3 Fig — Anti-CD40 agonist antibody was injected to C57BL/6 mice to induce inflammation, and disease induction was evaluated based on body weight change (A), colonoscopy at Day 3 and Day 6 post-anti-CD40 injection (B), percent of IBA1+ and CD3+ area of total mucosal area, mucosal thickness as well as percent of goblet cell area of total mucosal area (C), and upregulation of CD86 expression in splenic B and T cells (D). In (B), representative images for Day 6 colonoscopy are shown. In (C), representative images for Day 7 histology are shown. Scale bar, 100μM. * = P<0.001 Data are representative results from two independent experiments. (TIF) [file pone.0228221.s003.tif]
